# Long-term dietary fiber intake and risk of chronic obstructive pulmonary disease: a prospective cohort study of women

**DOI:** 10.1007/s00394-019-02038-w

**Published:** 2019-07-06

**Authors:** Maria Karolina Szmidt, Joanna Kaluza, Holly Ruth Harris, Anders Linden, Alicja Wolk

**Affiliations:** 1 Department of Human Nutrition, Warsaw University of Life Sciences-SGGW, 159C Nowoursynowska Str., 02-776 Warsaw, Poland; 2 Unit of Cardiovascular and Nutritional Epidemiology, Institute of Environmental Medicine, Karolinska Institutet, 171-77 Stockholm, Sweden; 3 Program in Epidemiology, Division of Public Health Sciences, Fred Hutchinson Cancer Research Center, Seattle, WA USA; 4 Department of Epidemiology, University of Washington, Seattle, WA USA; 5 Unit for Lung and Airway Research, Institute of Environmental Medicine, Karolinska Institutet, 171-77 Stockholm, Sweden; 6 Department of Respiratory Medicine and Allergy, New Karolinska Solna, Karolinska University Hospital, 171-77 Stockholm, Sweden; 7 Department of Surgical Sciences, Uppsala University, Uppsala, Sweden

**Keywords:** Chronic obstructive pulmonary disease, Diet, Dietary fiber, Epidemiology, Prospective cohort study

## Abstract

**Purpose:**

Until now, only two prospective cohort studies have investigated dietary fiber intake in relation to risk of chronic obstructive pulmonary disease (COPD), but neither examined long-term fiber intake. Both studies reported that total fiber intake was associated with decreased COPD risk; however, results for specific fiber sources were inconsistent. Thus, we prospectively evaluated the association between baseline and long-term intake of dietary fiber and COPD risk in a population-based prospective cohort of 35,339 Swedish women.

**Methods:**

Dietary fiber intake was assessed in 1987 and 1997 with a food frequency questionnaire. Cox proportional hazard regression models were used to estimate hazard ratios (HRs) and 95% confidence intervals (CIs).

**Results:**

During follow-up (2002–2014), 1557 COPD cases were identified via linkage to the Swedish National Patient Register. Long-term high dietary fiber intake (≥ 26.5 vs. < 17.6 g/day) was associated with a 30% (95% CI 17–41%) lower risk of COPD. For specific fiber sources, cereal (≥ 16.3 vs. < 9.4 g/day; HR 0.67, 95% CI 0.55–0.81) and fruit fiber (≥ 7.6 vs. < 2.6 g/day; HR 0.65, 95% CI 0.5–0.81), but not vegetable fiber intake (≥ 5.4 vs. < 2.2 g/day; HR 1.03, 95% CI 0.81–1.28) were associated with lower COPD risk. Current and ex-smokers with low long-term total fiber intake (< 17.6 g/day) compared to never smokers with high intake (**≥ **26.5 g/day) had a 33-fold (95% CI 23.6–46.6) and tenfold (95% CI 7.0–16.3), respectively, higher risk of COPD.

**Conclusions:**

Our findings indicate that high fiber intake is a modifiable lifestyle factor which may decrease COPD risk primarily in current and ex-smokers.

**Electronic supplementary material:**

The online version of this article (10.1007/s00394-019-02038-w) contains supplementary material, which is available to authorized users.

## Introduction

Globally, chronic obstructive pulmonary disease (COPD) is predicted by the World Health Organisation to become the third most common disease-related cause of mortality by the year 2030 [1]. Exposure to noxious particles or gases which cause airway and/or alveolar abnormalities leads to COPD development; and tobacco smoking is COPD’s main risk factor [1]. COPD is characterized by chronic inflammation which is usually initiated locally in the airways, but as the disease progresses, it becomes systemic [1, 2]. It has been observed that, especially among smokers [3], chronically elevated serum concentrations of C-reactive protein (CRP) [4] and interleukin-6 (IL-6) [5] are associated with a loss of lung function and higher risk of COPD. Unfortunately, the efficacy of current pharmacotherapy against COPD remains poor; thus, it is important to seek modifiable lifestyle factors which could reduce risk of COPD. The health benefits of dietary fiber in relation to inflammatory diseases such as type 2 diabetes [6], metabolic syndrome [7] and cardiovascular diseases [8] have been well documented. Thus, it is feasible that dietary fiber intake, through anti-inflammatory [9–11] as well as indirect antioxidant properties [12], may protect lungs against inflammation and prevent COPD, especially through modulation of the innate immune system via the gut–liver–lung axis [13] and enhancing the bioavailability of antioxidants [14].

Until now, only two prospective cohort studies have investigated dietary fiber intake in relation to risk of COPD development. The first was conducted in two cohorts of US women and men [15] and the second in a cohort of Swedish men [16]. Results of these studies indicate that total as well as cereal dietary fiber are inversely associated with COPD incidence, while results obtained for fruit and vegetable fiber intake were inconsistent [15, 16]. Fruit and vegetable fiber has been inversely associated with COPD incidence only among current smokers in the cohort of Swedish men [16]. Moreover, no previous studies have assessed the risk of COPD in relation to long term, in contrast to only baseline, dietary fiber intake.

In the current study, we hypothesized that intake of dietary fiber is a beneficial lifestyle choice that reduces the risk of COPD. To address this hypothesis, we investigated the associations between long-term total and specific fiber intake and incidence of COPD in the population-based prospective Swedish Mammography Cohort.

## Methods

### Study population

**Fig. 1:**
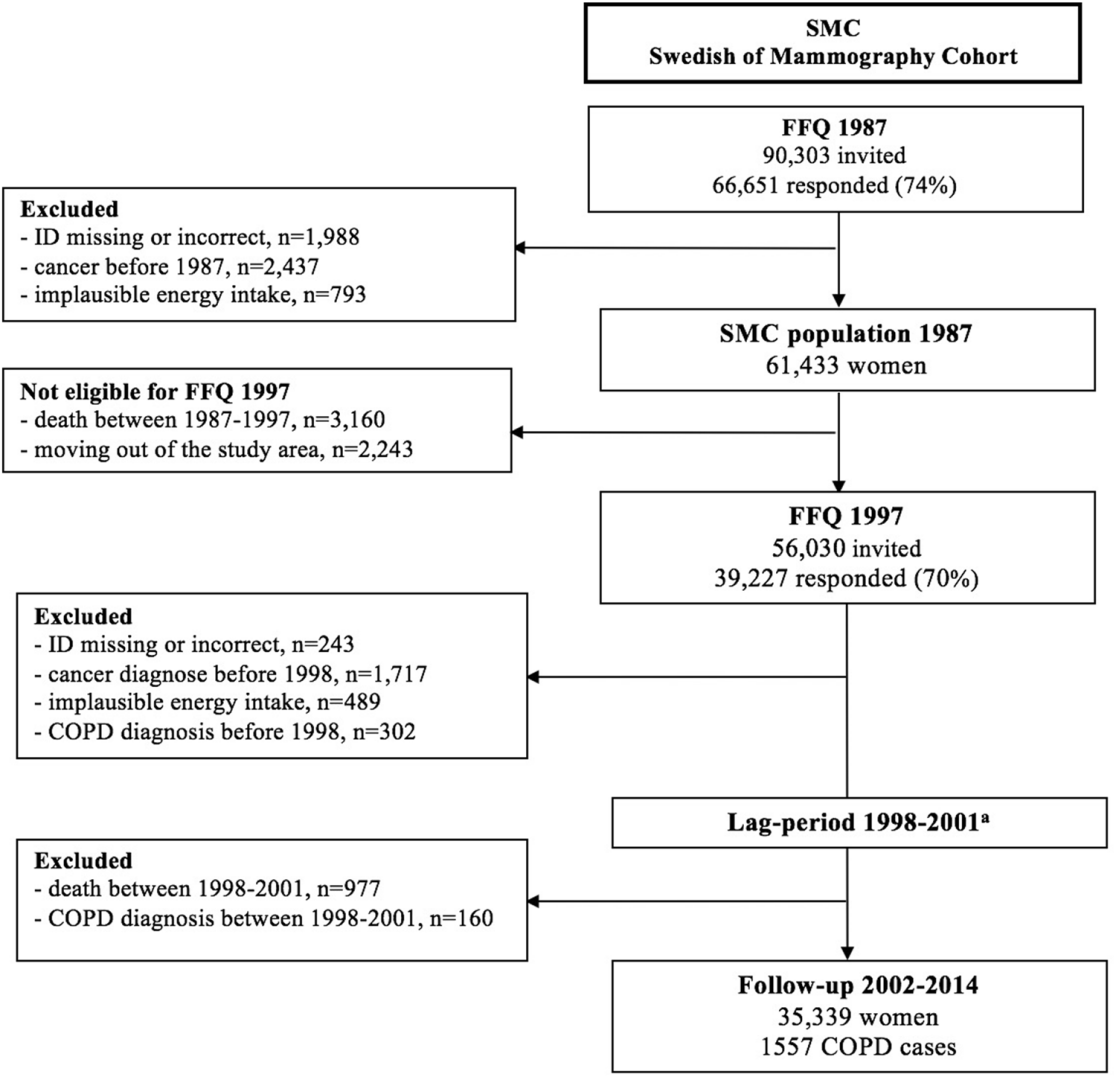
Flow chart of the study population. (Superscript a) A 4-year lag period was introduced due to COPD under-diagnosis between 1998 and 2001 (details in online Supplementary Material and Supplementary Table S2)

The Swedish Mammography Cohort (SMC) was established between 1987 and 1990 when all women living in Uppsala and Västmanland counties in central Sweden, and born during 1914–1948 (*n* = 90,303) were invited to a mammography screening and completed a food-frequency questionnaire. Among the 66,651 (response rate = 74%) women who returned the questionnaire, we excluded those with missing or incorrect national identification number (*n* = 1988), history of cancer (other than non-melanoma skin cancer, *n* = 2437), and improbable total energy (± 3 SDs from the log-transformed mean energy intake, i.e., daily intake < 575 kcal and > 4664 kcal, *n* = 793) (Fig. 1). After these exclusions, the baseline prospective cohort consisted of 61,433 women. Ten years later, in 1997, a second expanded questionnaire was sent to all of the women who were still alive and were living in the same region of Sweden (*n* = 56,030). The completed questionnaires were received from 39,227 women (response rate = 70%). From these women, we excluded those with missing or incorrect national identification number (*n* = 243), who were diagnosed with cancer (*n* = 1717) or COPD before 1998 (ICD-10 code: J44, *n* = 302), and those with improbable total energy intake (*n* = 489). Additionally, due to a potential under-diagnosis in the first years of follow-up (1998–2001) and the necessity of a 4-year lag period introduction (details in the “Case ascertainment” section and in online Supplementary Material—Figure S1), women with COPD diagnosis between 1998 and 2001 (*n* = 160) or those who died during these 4 years (*n* = 977) were excluded as well. Thus, the final analytic cohort consisted of 35,339 women, aged 61.5 ± 9.1 years, who were comparable to the general Swedish female population in relation to age distribution, education, and BMI distribution [17]. In the present study, the data collected in 1997 were used as baseline because information on some important COPD risk factors, such as smoking and physical activity, was not obtained in 1987.

### Assessment of dietary fiber intake

Data about food consumption were collected by food frequency questionnaires (FFQs) based on 67 food items in 1987 and 96 food items in 1997. Participants were asked about their average food consumption during the past year based on eight predefined frequency categories, ranging from “never/seldom” to “4 times per day” in 1987, and from “0 times per month” to “3 + times per day” in 1997. Dietary fiber intake was estimated by multiplying the frequency of intake of each food item by the fiber content (based on data from Swedish National Administration Database) [18] using age-specific portion sizes. Specific food items used in the calculation of dietary fiber intake are presented in Supplementary Table S1. Intake of cereal, fruit, and vegetable fiber was determined by summing the fiber components of individual food items that contributed to intake of each type of fiber. To minimize the effects of measurement error in dietary fiber intake, the residual method was used. Through this procedure “energy-adjusted” fiber intake was computed as the residual from the regression model using total caloric intake as the independent variable and absolute fiber intake as the dependent variable [19]. Using this method, fiber intake was adjusted for total energy intake to 1600 kcal/day in 1987 and to 1700 kcal/day in 1997 (the mean energy intake in both diet measurements) [20]. To calculate long-term dietary fiber intake, the cumulative average method was used based on data collected in 1987 (10 years prior baseline) and 1997 (baseline). This approach represents the most stable estimate of fiber intake and better represent the long-term dietary intake [21]. The 1997 FFQ has been validated among 248 study participants (aged 40–74 years old) in central Sweden, by comparing with fourteen 24-h recall interviews [22]. The Spearman correlation coefficient between the two methods for dietary fiber was 0.71.

### Assessment of other exposures

In the present study, information on covariates collected on the 1997 questionnaire was used as baseline. Women were asked about education level, weight, height, time per day spent on walking and/or cycling, smoking status, and alcohol consumption. Body Mass Index (BMI) was calculated by dividing the weight in kilograms by the square of the height in meters. Participants reported average number of cigarettes smoked per day at different ages (15–20, 21–30, 31–40, 41–50, 51–60 years old, and in the year of data collection), and indicated when they started and stopped smoking (only if this event occurred). Pack-years of smoking were calculated by multiplying the number of cigarettes smoked per day by number of years smoked, respectively, for each age category.

### Case ascertainment

Linkage to the Swedish National Patient Registers (inpatient and outpatient) and the Swedish Cause of Death Register was performed using the unique personal identification number assigned to each Swedish resident. COPD diagnoses were identified in accordance with the International Classification of Diseases and Related Health Problems, 10th Revision (ICD-10 code: J44). Incident COPD cases were defined as the first diagnosis of COPD in the Swedish Patient Register (listed either as the primary or any diagnosis position) or in the Cause of Death Register (only the primary position). Taking into account that both medical registers in Sweden and several guidelines for the diagnosis and management of the COPD were created in the early 90 s, there was a potential of under-diagnosis in the cohort [23, 24]. Furthermore, the results of a previous study reported that the proportion of COPD diagnoses increased from 59% in 1999 to 81% in 2009 in Sweden [23]. Thus, due to potential under-diagnosis of COPD in the study cohort, we analyzed the annual occurrence of COPD cases in 1998 and 2014 (see online Supplementary Figure S1). This analysis indicated that during the first 4 years of follow-up (1998–2001), the number of COPD cases was lower than after 2001 (40 vs. 119 COPD cases per year); thus, we implemented a 4-year lag period and started analyses from 2002.

### Statistical analysis

Women were followed from 1 January 2002 to 31 December 2014, and until COPD diagnosis, death, or the end of follow-up, whichever occurred first. Hazard ratios (HRs) and 95% confidence intervals (CIs) of COPD incidence were estimated using Cox proportional hazard regression models. Participants were assigned to quintiles of baseline energy-adjusted total dietary fiber intake (< 17.6, 17.6–20.5, 20.6–23.1, 23.2–26.4, and ≥ 26.5 g/day), and the same cut-points were used for long-term fiber intake. For specific fiber intake (both baseline and long term), participants were assigned to quintiles of energy-adjusted consumption. For all dietary fiber exposures, the lowest quintile served as the reference group. Multivariable models were adjusted for: age (years, continuous), education (less than high school, high school, university), BMI (< 18.5, 18.5–24.9, 25.0–29.9, ≥ 30 kg/m^2^), walking/cycling (< 20, 20–60, > 60 min/day), smoking status and pack-years of smoking (never; past < 20, 20–39, ≥ 40 pack-years; current < 20, 20–39, ≥ 40 pack-years), energy intake (kcal/day, quintiles), and alcohol intake (g/day, quintiles). For specific fiber sources, intakes of fiber from cereals, fruits, and vegetables were mutually adjusted by inclusion in the same multivariable model. Missing data on smoking status (1.8%), educational level (0.3%), BMI (1.7%), and walking/cycling (7.8%) were included as separate categories. Moreover, we investigated the combined effect of long-term total dietary fiber intake with smoking status (current, ex-, never smokers) where never-smoking women with the highest fiber intake were considered as the reference group.

To provide greater diagnostic power than unscaled residuals, the proportional hazards assumption was evaluated by regressing scaled Schoenfeld residuals against survival time, and evidence of rejection of the assumption was not found. To test linear trends, we used fiber intake as continuous variable. A likelihood ratio test was used to examine the interaction between long-term fiber intake and smoking status (ever vs. ex-smokers vs. current smokers) on COPD risk. The shape of the association between risk of COPD and long-term total dietary fiber intake was investigated using restricted cubic-splines with three knots (at the 10th, 50th, 90th percentile) [25]. This method allows the examination of the effect of a continuous predictor (long-term total dietary fiber intake) on an outcome (COPD incident) reducing model misspecification and providing insight into the relationship between long-term fiber intake and COPD. Statistical analyses were conducted using SAS v. 9.4. (SAS Institute Inc, Cary, NC, USA) and STATA v. 13 (StataCorp, College Station, TX, USA). All *P* values were two-sided and values ≤ 0.05 were considered statistically significant.

## Results

### Characteristics of the cohort

In 1997, the energy-adjusted mean total dietary fiber intake was 22.2 ± 5.5 (median 21.8) g/day and the contribution of cereal, fruit and vegetables to total fiber intake was 59.1%, 23.2%, 17.5%, respectively. The Spearman correlation coefficients between total fiber intake and cereal, fruit, and vegetable fiber were 0.57, 0.53, and 0.51, respectively. Compared to women in the lowest quintile of total dietary fiber intake (< 17.6 g/day), those in the highest quintile (≥ 26.4 g/day) were less likely to be ever smokers and more likely to walk and/or cycle more than 20 min/day (Table 1). Women with the highest intake of dietary fiber compared to those with the lowest intake had, on average, more than twofold higher consumption of whole-grain products, fruits, and vegetables, while consuming less processed red meat and alcohol. In 1987, the energy-adjusted mean total dietary fiber intake was 24.4 ± 5.5 (median 24.1) g/day. Data on intake of dietary fiber collected using the 1987 FFQ and 1997 FFQ were characterized by high agreement (illustrated with the Bland–Altman plot in online Supplementary Figure S2).

**Table 1:** Age-standardized characteristics of 35,339 women in the Swedish Mammography Cohort (SMC) by quintiles of energy-adjusted total dietary fiber intake at baseline (1997)

	Quintiles of dietary fiber intake, g/day (median)^a^					*P* value^b^
	< 17.6 (15.6)	17.6–20.5 (19.2)	20.6–23.1 (21.8)	23.2–26.4 (24.6)	≥ 26.5 (29.1)	
*N*	7016	7073	7072	7091	7087	
Age, years	60.6 ± 9.4^c^	61.1 ± 9.1	61.8 ± 9.1	62.0 ± 9.0	62.0 ± 9.0	< 0.001
Fiber intake in 1987, g/day^d^	21.0 (6.6)^e^	23.0 (6.1)	24.2 (6.1)	25.5 (6.4)	27.0 (7.1)	< 0.001
Education, university, %	17.6	19.2	19.3	19.6	19.0	0.01
Body Mass Index, kg/m^2^	24.9 ± 4.0	25.0 ± 3.9	25.0 ± 3.9	25.1 ± 3.9	25.2 ± 4.0	< 0.001
Walking/cycling, %						
< 20 min/day 20–60 min/day > 60 min/day	33.8 44.0 13.3	28.6 49.9 14.1	26.7 50.1 15.9	24.8 51.6 16.7	21.7 50.7 19.4	0.12
Smoking status, %						
Current smokers Ex-smokers Never smokers	30.3 21.1 46.6	23.6 22.3 52.5	21.1 23.0 54.2	18.8 23.0 56.5	18.7 24.3 55.1	0.84
Alcohol consumption, g/day	5.3 ± 7.0	4.6 ± 5.4	4.2 ± 4.9	3.7 ± 4.2	3.1 ± 3.9	< 0.001
Energy intake, kcal/day	1750 ± 564	1752 ± 522	1737 ± 501	1727 ± 498	1725 ± 539	< 0.001
Whole-grain products, g/day	61.0 ± 60.0	82.6 ± 68.8	96.5 ±73.8	111 ± 84.5	127 ± 88.0	< 0.001
Fruits, g/day	107 ± 74	150 ± 89	181 ± 104	217 ± 122	291 ± 181	< 0.001
Vegetables, g/day	125 ± 72	159 ± 82	186 ± 95	214 ± 107	289 ± 186	< 0.001
Processed red meat, g/day	31.8 ± 29.2	31.1 ± 23.9	30.8 ± 25.5	29.0 ± 24.3	25.1 ± 24.0	< 0.001

^a^Energy-adjusted to 1700 kcal/day (mean energy intake in the study cohort based on FFQ 1997) using the residual method

^b^*P* values were calculated across quintiles of dietary fiber intake by using age-adjusted linear models for continuous variable and Pearson’s Chi square for categorized variables

^c^Mean ± SD—all such values

^d^Energy-adjusted to 1600 kcal/day (mean energy intake in the study cohort based on FFQ 1987) using the residual method

^e^Median (interquartile range)

### Baseline fiber intake

During a mean follow-up of 11.5 years (407,067 person-years, 2002–2014), 1557 incident cases of COPD were identified. An inverse association between total baseline dietary fiber intake and risk of COPD development was observed (Table 2). Women in the highest quintile of total dietary fiber intake (≥ 26.5 g/day) compared to those in the lowest quintile (< 17.6 g/day) had a 22% (95% CI 9–33%) lower risk of COPD. Each 1-g increment in total fiber intake (up to 25 g/day) was associated with 3% (95% CI 2–5%) risk reduction of COPD. For specific fiber sources, we observed that cereal fiber and fruit fiber intake were inversely associated with risk of COPD (Table 2). The HRs between women in the highest and lowest quintiles of cereal fiber and fruit fiber were 0.84 (95% CI 0.72–0.98) and 0.77 (95% CI 0.65–0.91), respectively. No association was observed for vegetable fiber intake and risk of COPD.

**Table 2:** Hazard ratios (95% CIs) of chronic obstructive pulmonary disease by quintiles of energy-adjusted baseline dietary fiber intake (1997) in 35,339 Swedish women, follow-up 2002–2014

	Quintiles of dietary fiber intake, g/day (median)^a^					Dietary fiber intake up to 25 g/day^a^	
						Per 1-g	*P* trend
Total fiber intake	< 17.6 (15.6)	17.6–20.5 (19.2)	20.6–23.1 (21.8)	23.2–26.4 (24.6)	≥ 26.5 (29.1)		
No. of cases/no. women	446/7016	314/7073	290/7072	248/7091	259/7087		
Age-adjusted HR (95% CI)	1.00	0.67 (0.58–0.77)	0.61 (0.53–0.71)	0.51 (0.44–0.59)	0.53 (0.46–0.62)	0.92 (0.91–0.94)	< 0.001
Age and smoke-adjusted HR (95% CI)	1.00	0.81 (0.70–0.94)	0.80 (0.69–0.93)	0.72 (0.61–0.84)	0.77 (0.66–0.90)	0.96 (0.95–0.97)	< 0.001
Multivariable-adjusted HR (95% CI)^b^	1.00	0.84 (0.73–0.98)	0.83 (0.72–0.97)	0.74 (0.63–0.87)	0.78 (0.67–0.91)	0.97 (0.95–0.98)	< 0.001
Cereal fiber	< 9.4 (7.9)	9.4–11.6 (10.6)	11.7–13.5 (12.5)	13.6–16.2 (14.7)	≥ 16.3 (18.6)		
No. of cases/no. women	372/7261	306/7261	268/7261	282/7261	329/7261		
Multivariable-adjusted HR (95% CI)^b^	1.00	0.89 (0.76–1.03)	0.80 (0.68–0.93)	0.83 (0.71–0.97)	0.84 (0.72–0.98)	0.98 (0.96–1.00)	0.01
Fruit fiber	< 2.6 (1.8)	2.6–4.0 (3.2)	4.1–5.3 (4.6)	5.4–7.5 (6.3)	≥ 7.6 (9.4)		
No. of cases/no. women	497/7244	369/7278	242/7261	221/7261	228/7261		
Multivariable-adjusted HR (95% CI)^b^	1.00	1.00 (0.87–1.14)	0.78 (0.66–0.90)	0.73 (0.61–0.86)	0.77 (0.65–0.91)	0.95 (0.92–0.97)	< 0.001
Vegetable fiber	< 2.2 (1.6)	2.2–3.0 (2.6)	3.1–3.9 (3.5)	4.0–5.3 (4.5)	≥ 5.4 (6.7)		
No. of cases/no. women	411/7261	317/7261	282/7261	243/7261	304/7261		
Multivariable-adjusted HR (95% CI)^b^	1.00	0.92 (0.79–1.07)	0.90 (0.77–1.05)	0.81 (0.69–0.96)	0.97 (0.83–1.13)	0.97 (0.94–1.01)	0.08

*HR* hazard ratio, *CI* confidence interval

^a^Energy-adjusted to 1700 kcal/day (mean energy intake in the study cohort based on FFQ 1997) using the residual method

^b^Adjusted for age (continuous), education (less than high school, high school, or university), BMI (< 18.5; 18.5–24.9; 25.0–29.9, or ≥ 30.0 kg/m^2^), walking or cycling (< 20, 20–60, > 60 min/day), smoking status and pack-years of smoking (never; past < 20, 20–39, or ≥ 40 pack-years; or current < 20, 20–39, or ≥ 40 pack-years), intake of energy (kcal/day, quintiles) and alcohol consumption (g/day, quintiles)

Due to the observed under-diagnosis of COPD incidence from 1998–2001, we performed a sensitivity analysis and calculated HRs stratified by time of diagnosis (see online Supplementary Table S2). The HRs in years 2002–2008 and 2009–2014 were consistent; between extreme quintiles of total fiber intake (≥ 26.5 vs. < 17.6 g/day) were 0.75 (95% CI 0.60–0.94) in 2002–2008 and 0.81 (95% CI 0.65–1.00) in 2009–2014. In 1998–2001, a statistically significant association between dietary fiber intake and COPD risk was not observed (HR 1.02, 95% CI 0.64–1.63). Taking into account the lower number of identified COPD cases in 1998–2001, we hypothesized that the lack of association was at least partly the result of under-diagnosis of COPD incidence during that period.

### Long-term fiber intake

**Fig. 2:**
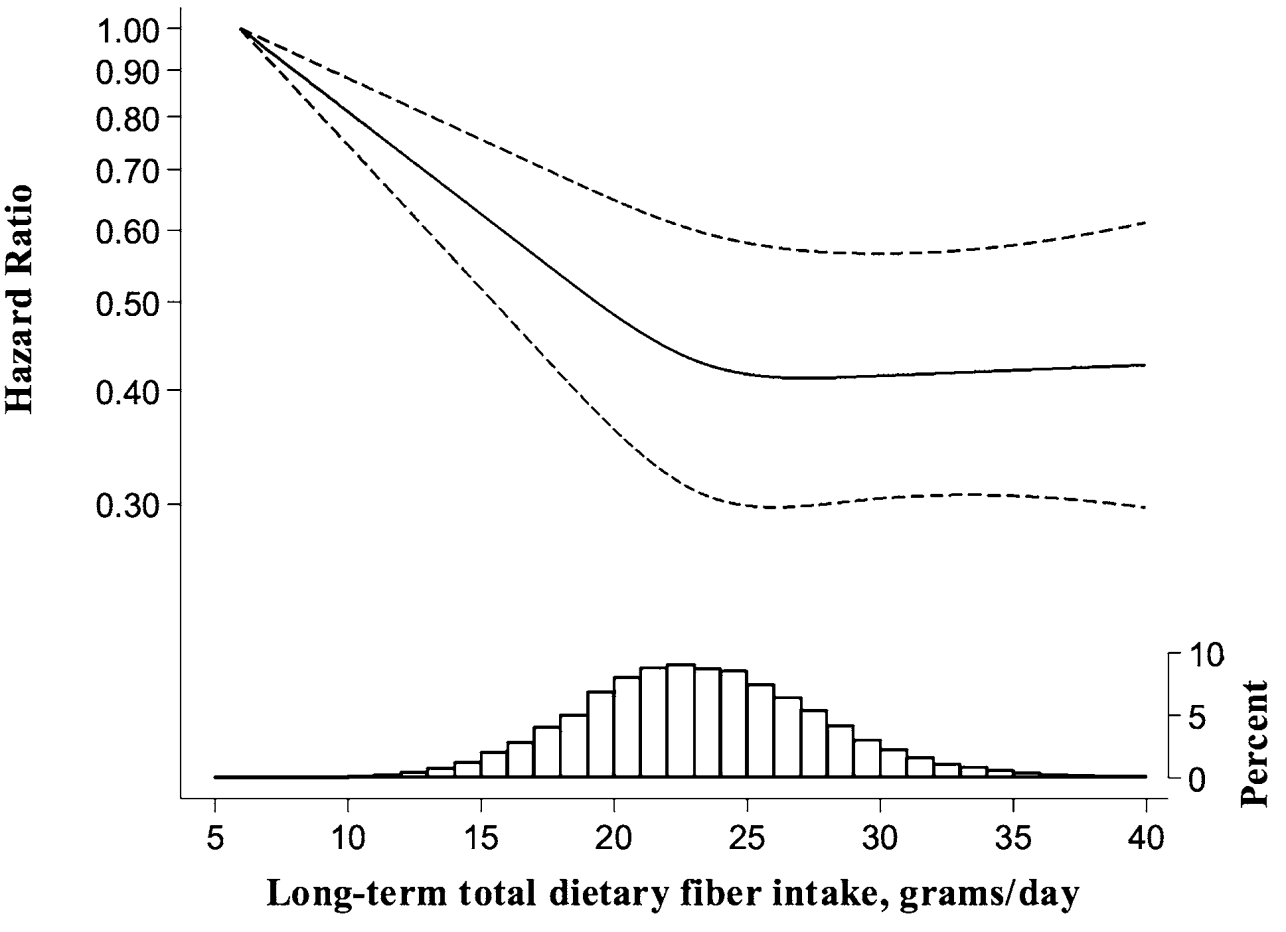
Multivariable-adjusted hazard ratios (solid line) of COPD incidence as a function of long-term total dietary fiber intake among Swedish women (*P* value for non-linearity = 0.005). The hazard ratio is presented on the log scale. The long-dashed lines represent 95% confidence intervals. The distribution of long-term total dietary fiber intake is presented at the bottom of the figure as a histogram

Taking into consideration long-term dietary fiber intake, reflecting the average intake at baseline (1997) and 10 years before baseline (1987), women in the highest vs. lowest quintiles of fiber intake had a 30% (95% CI 17–41%) lower risk of COPD (Table 3). Using a restricted cubic spline analysis, we assessed the shape of the association between risk of COPD and long-term total dietary fiber intake, and observed a non-linear association, *P* value for non-linearity = 0.005 (Fig. 2). The risk of COPD decreased with increasing total dietary fiber intake up to 25 g/day, with each 1 g/day increment associated with 5% (95% CI 3–7%) risk reduction, without a further decrease in risk with increasing intake.

**Table 3:** Hazard ratios (95% CIs) of chronic obstructive pulmonary disease by energy-adjusted long-term dietary fiber intake (data from 1987 to 1997) in 35,339 Swedish women, follow-up 2002–2014

	Long-term dietary fiber intake, g/day (median)^a^					Long-term dietary fiber intake up to 25 g/day^a^	
						Per 1-g	*P* trend
Total fiber	< 17.6 (16.1)	17.6–20.5 (19.3)	20.6–23.1 (21.8)	23.2–26.4 (24.6)	≥ 26.5 (28.6)		
No. of cases/no. women	267/3528	307/6149	363/8014	307/9384	313/8264		
Age-adjusted HR (95% CI)	1.00	0.62 (0.52–0.73)	0.54 (0.46–0.63)	0.37 (0.32–0.44)	0.43 (0.36–0.50)	0.89 (0.88–0.91)	< 0.001
Age and smoke-adjusted HR (95% CI)	1.00	0.77 (0.65–0.90)	0.77 (0.65–0.90)	0.57 (0.48–0.67)	0.69 (0.58–0.81)	0.94 (0.93–0.96)	< 0.001
Multivariable-adjusted HR (95% CI)^b^	1.00	0.80 (0.67–0.94)	0.81 (0.69–0.95)	0.60 (0.51–0.71)	0.70 (0.59–0.83)	0.95 (0.93–0.97)	< 0.001
Cereal fiber	< 9.4 (8.4)	9.4–11.6 (10.7)	11.7–13.5 (12.6)	13.6–16.2 (14.8)	≥ 16.3 (18.3)		
No. of cases/no. of women	136/2021	237/4781	292/7120	409/10,294	483/11,123		
Multivariable-adjusted HR (95% CI)^b^	1.00	0.80 (0.64–0.99)	0.72 (0.59–0.89)	0.69 (0.56–0.84)	0.67 (0.55–0.81)	0.96 (0.94–0.98)	< 0.001
Fruit fiber	< 2.6 (1.9)	2.6–4.0 (3.3)	4.1–5.3 (4.6)	5.4–7.5 (6.2)	≥ 7.6 (10.0)		
No. of cases/no. of women	591/8185	410/9148	259/8204	198/6414	99/3388		
Multivariable-adjusted HR (95% CI)^b^	1.00	0.88 (0.77–1.00)	0.70 (0.60–0.81)	0.68 (0.57–0.80)	0.65 (0.52–0.81)	0.90 (0.87–0.94)	< 0.001
Vegetable fiber	< 2.2 (1.6)	2.2–3.0 (2.6)	3.1–3.9 (3.4)	4.0–5.3 (4.4)	≥ 5.4 (6.2)		
No. of cases/no. of women	679/13,492	378/9686	243/6339	171/3856	86/1966		
Multivariable-adjusted HR (95% CI)^b^	1.00	0.98 (0.86–1.12)	1.04 (0.90–1.22)	1.16 (0.97–1.38)	1.03 (0.81–1.28)	0.96 (0.91–1.01)	0.14

*HR* hazard ratio, *CI* confidence interval

^a^Long-term consumption was calculated as a cumulative average of energy-adjusted dietary fiber intake from 1987 to 1997

^b^Adjusted for age (continuous), education (less than high school, high school, or university), BMI (< 18.5; 18.5–24.9; 25.0–29.9, or ≥ 30.0 kg/m^2^), walking or cycling (< 20, 20–60, > 60 min/day), smoking status and pack-years of smoking (never; past < 20, 20–39, or ≥ 40 pack-years; or current < 20, 20–39, or ≥ 40 pack-years), intake of energy (kcal/day, quintiles) and alcohol consumption (g/day, quintiles)

Due to the potential importance of a woman’s height in airflow from the lungs, we performed a sensitivity analysis replacing the BMI categories with quintiles of height and weight in the model. The results did not differ with a HR of 0.70 (95% CI 0.59–0.82) comparing women in the highest vs. the lowest total dietary fiber intake.

For specific fiber sources, we observed an inverse association between risk of COPD and long-term cereal and fruit fiber intake (Table 3). Women with the highest cereal fiber intake and fruit fiber intake, compared to those with the lowest intake, had a 33% (95% CI 19–45%) and 35% (95% CI 19–48%) lower risk of COPD, respectively. No association was observed for vegetable fiber intake (Q5 vs. Q1, HR 1.03, 95% CI 0.81–1.28).

### Findings by smoking status

As smoking is the main risk factor for developing COPD, we conducted stratified analysis by smoking status (Table 4). Current and ex-smokers in the highest vs. lowest quintile of long-term fiber intake had a 26% (95% CI 9–40%) and 36% (95% CI 7–56%) lower risk of COPD, respectively. A similar suggestion also was observed in never smokers, with HR 0.66 (95% CI 0.37–1.19). In the dose–response analysis, each 1-g increment in long-term dietary fiber intake up to 25 g/day was associated with 5% (95% CI 2–7%) decreased risk in current smokers, 7% (95% CI 3–11%) in ex-smokers, and 3% in never smokers (95% CI − 4 to 9%). However, no statistically significant interaction was observed between long-term dietary fiber intake and smoking status (current vs. ex-smokers vs. never smokers *P* interaction = 0.79).

**Table 4:** Hazard ratios (95% CIs) of chronic obstructive pulmonary disease by energy-adjusted long-term total dietary fiber intake (data from 1987 to 1997) stratified by smoking status in Swedish women, follow-up 2002–2014

	Long-term total dietary fiber intake, g/day (median)^a^					Long-term dietary fiber intake up to 25 g/day^a^	
	< 17.6 (16.1)	17.6–20.5 (19.3)	20.6–23.1 (21.8)	23.2–26.4 (24.6)	≥ 26.5 (28.6)	Per 1-g	*P* trend
Current smokers							
No. of cases/no. of women	203/1259	221/1706	235/1770	174/1774	187/1449		
Multivariable-adjusted HR (95% CI)^b^	1.00	0.81 (0.67–0.99)	0.81 (0.67–0.98)	0.56 (0.46–0.69)	0.74 (0.60–0.91)	0.95 (0.93–0.98)	< 0.001
Ex-smokers							
No. of cases/no. of women	46/796	55/1370	78/1823	73/2085	78/1920		
Multivariable-adjusted HR (95% CI)^b^	1.00	0.71 (0.48–1.06)	0.78 (0.54–1.13)	0.58 (0.40–0.84)	0.64 (0.44–0.93)	0.93 (0.89–0.97)	< 0.001
Never smokers							
No. of cases/no. of women	16/1412	29/2957	48/4295	55/5356	42/4731		
Multivariable-adjusted HR (95% CI)^c^	1.00	0.82 (0.45–1.52)	0.89 (0.51–1.58)	0.79 (0.45–1.39)	0.66 (0.37–1.19)	0.97 (0.91–1.04)	0.39

*HR* hazard ratio, *CI* confidence interval

^a^Long-term consumption was calculated as a cumulative average of energy-adjusted dietary fiber intake from 1987 to 1997

^b^Adjusted for age (continuous), education (less than high school, high school, or university), BMI (< 18.5; 18.5–24.9; 25.0–29.9, or ≥ 30.0 kg/m^2^), walking or cycling (< 20, 20–60, > 60 min/day), smoking status and pack-years of smoking (never; past < 20, 20–39, or ≥ 40 pack-years; or current < 20, 20–39, or ≥ 40 pack-years), intake of energy (kcal/day, quintiles) and alcohol consumption (g/day, quintiles)

^c^Adjusted as above with exception pack-years of smoking

**Fig. 3:**
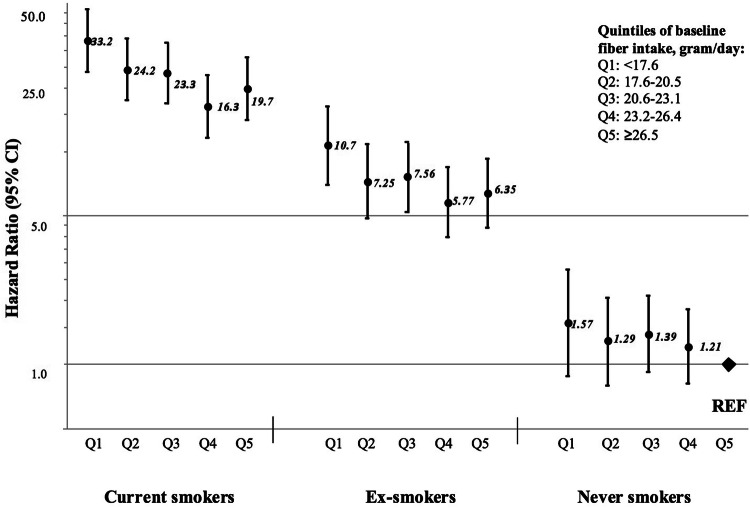
Hazard ratios (95% confidence intervals) of chronic obstructive pulmonary disease by long-term energy-adjusted total dietary fiber intake and smoking status in Swedish women (*P* value for interaction = 0.52), follow-up 2002–2014

To investigate the combined effect of long-term dietary fiber intake and smoking status on risk of COPD, a comprehensive analysis which included current, ex-smokers and never smokers in one model was performed (Fig. 3). In comparison to never smokers in the highest quintile of fiber intake (≥ 26.5 g/day), current and ex-smokers with the lowest intake (< 17.6 g/day) had a 33-fold (HR 33.2, 95% CI 23.6–46.6) and 11-fold (HR 10.7, 95% CI 7.0–16.3) higher risk of COPD, respectively. With increasing long-term dietary fiber intake, risk of COPD was decreased to 20-fold (HR 19.7, 95% CI 14.1–27.5) for current smokers and sixfold (HR 6.4, 95% CI 4.4–9.3) for ex-smokers.

## Discussion

In this prospective cohort study of women, we found that high dietary fiber intake was inversely associated with the risk of COPD, and the risk was decreased in all categories of smoking status—current and ex-smokers, as well as never smokers. For specific fiber sources, cereal and fruit fiber, but not vegetable fiber, was inversely associated with COPD incidence.

Our findings confirm and expand upon the results from two previous prospective cohort studies (US-based study including 40,215 men and 71,365 women, 832 COPD cases [15] and Swedish study including 45,058 men, 1982 COPD cases [16]) which indicated that total intake of dietary fiber was inversely associated with risk of COPD. Those studies observed that participants in the highest vs. the lowest quintile of dietary fiber intake had a 33% (95% CI 10–50%, *P* trend = 0.03) [15] and 38% (95% CI 29–47%, *P* trend < 0.0001) [16] lower risk of COPD, respectively. Our findings are also in line with a previous cross-sectional study which investigated associations between total dietary fiber intake and lung function among 11,897 US participants [26], where higher fiber intake was associated with higher forced expiratory volume in 1 s (FEV_1_), forced vital capacity (FVC) and FEV_1_/FVC ratio.

Moreover, some previous studies have examined the associations between dietary patterns and risk of COPD. A “Prudent” dietary pattern (rich in whole grains, fruits, vegetables, fish, poultry) which is characterized by high amounts of dietary fiber, was inversely associated with COPD risk [27–29]. Moreover, results of those studies showed that a “Western” dietary pattern (rich in refined grains, sugar, cured and red meat, French-fries), which contains low amounts of fiber, was positively associated with risk of COPD [27–29]. Furthermore, participants with the highest vs. the lowest degree of “Western” dietary pattern had lower FEV_1_, FVC and FEV_1_/FVC ratio, while those with the highest vs. the lowest “Prudent” dietary pattern had higher values of spirometry parameters [27]. Results of these studies indicate that the presence of healthy diet (“Prudent” dietary pattern) and absence of unhealthy diet (“Western” dietary pattern) may be associated with a lower risk of COPD.

Considering sources of dietary fiber intake, our results confirmed findings of previous studies that higher consumption of cereal fiber was associated with lower risk of COPD [15, 16]. Among US participants, cereal fiber was inversely associated with lower risk of COPD, while similar associations were not observed for fruit and vegetable fiber [15]. Among Swedish men, cereal and fruit fiber was associated with lower risk in current and ex-smokers, while vegetable fiber was inversely associated only in current smokers [16]. The lack of an inverse association between vegetable fiber intake and COPD incidence could be explain by the higher uptake of heavy metals, especially cadmium and lead, in vegetables compared to fruits [30, 31]. Among smokers who are already exposed to toxic substances in the form of cigarette smoke, including heavy metals, each additional source of harmful substances (e.g., cadmium and lead in vegetables), could add to the adverse effects in the lung caused by smoking. Moreover, vegetables contain higher amounts of nitrate than fruits, and the source about 80% of this form of nitrogen in the usual diet is raw vegetables [32]. In 2008, the European Food Safety Authority stated that the conversion of nitrate into nitrite in vegetables (by salivary enzymes and oral bacteria or during storage) may contribute to adverse health effects [33]. Nitrate metabolites and their reaction products (e.g., peroxynitrite) provoke inflammatory processes in the airway and lung parenchyma, impairing respiratory homeostasis and causing cell damage [34].

In contrast to a previous study of Swedish men [16], we did not observe a significant interaction between dietary fiber intake and smoking status in relation to risk of COPD in Swedish women. However, the results for current and ex-smokers in the current study are in line with those obtained for men [16]. The observed risk reduction for total dietary fiber intake was higher among men who were current smokers compared to women who were current smokers; while among ex-smokers, the results were quite similar for men and women [16]. The observed differences between current smoking between men and women may be due to the amounts of total dietary fiber intake and contribution of different fiber sources. Total dietary fiber intake was 1.5-fold higher among men [16] than women (medians Q1–Q5 were 20.5–40.9 g/day in men, and 16.1–28.6 g/day in women), and the contribution of cereal fiber to total fiber intake was 67% in men and 59% in women, fruit fiber was 12% in men and 23% in women, while vegetable fiber was 9% and 18%, respectively. Moreover, differences between results obtained for Swedish women and men [16] could be related to differences in smoking intensity and smoking pack-years within these two cohorts. Median pack-years of smoking was higher among men (18.1 pack-years) compared to women (12.9 pack-years). Furthermore, women may have a different sensitivity to harmful components present in cigarette smoke, and it may be a result of sex-specific pathogenic mechanisms, sex hormones, or different baseline health status of men and women.

Various potential mechanisms involving a role of dietary fiber in the modulation of systemic tissue inflammation may explain the inverse association between dietary fiber intake and risk of COPD. Prior studies have demonstrated that higher dietary fiber intake decreased the concentration of pro-inflammatory mediators such as IL-6 and CRP [9–11]. Results from two US studies have showed that participants with the highest vs. the lowest total fiber intake had a 63% and 32% lower risk of high concentrations of CRP and IL-6, respectively. It has also been reported that dietary fiber may lower risk of COPD through modulating the innate immune system via the gut–liver–lung axis [13]. This mechanism focuses on the impact of dietary fiber on the gut microbiome, which plays a key role in the function of the host immune system [35]. One of the products of bacterial fermentation of fiber in the gut is the short-chain fatty acids (SCFA), which have been linked to immune response [36, 37]. Acetate, propionate and butyrate, synthesized in the bowel by beneficial bacteria through fermentation of insoluble fiber (present in high amounts in whole grains, nuts, some vegetables) [37, 38], are absorbed into the circulation and then can be used by liver to modify innate immune response. SCFA can modify immune response to pulmonary inflammation [39] through the activation of G-protein receptors on neutrophils and macrophages, inhibiting histone deacetylase, 3-hydroxy-3-methylglutaryl-coenzyme A reductase and nuclear factor-κB (NF-κB). These processes may result in decreased concentration of pro-inflammatory serum parameters such as IL-6 and CRP [40–42].

It seems feasible that dietary fiber may have a beneficial impact on reducing oxidative stress in the lungs via the antioxidant capacity of fruit and vegetables, thus, lowering the risk of COPD. Antioxidants such as polyphenols and carotenoids traverse the small intestine in conjunction with dietary fiber and may release the fiber matrix exclusively in the colon through the action of the microbiota [14]. Linkage to dietary fiber seems to be crucial for adequate assimilation of antioxidants in the gut [12, 14].

In light of the considerations above, dietary fiber seems to be a unique and important nutrient in the prevention of COPD, especially among smokers. Due to the modulation of the innate immune system via the gut–liver–lung axis and reduction in oxidative stress via enhancing bioaccessibility of antioxidants from fruits and vegetables, dietary fiber appears to be unique, even when compared to other nutrients with anti-inflammatory and anti-oxidative properties.

As strengths of this study, the following aspects should be considered: a large population-based prospective cohort and a large number of COPD cases which allowed sufficient power for comprehensive statistical analyses. This is the first study which examined long-term dietary fiber intake in relation to COPD incidence. Data on fiber intake had high validity [22]. Moreover, the available data on COPD risk factors allowed us to thoroughly adjust for potential confounders; however, some variables which are related to COPD development could not be included in the analysis (e.g. exposure to air pollution or occupational exposures). FFQ-based dietary data despite high validity could be misclassified between quintiles of total and specific sources of fiber intake. Furthermore, during the years of follow-up, diagnoses of COPD were based on post-bronchodilator values with a fixed FEV1/FVC ratio; however, some patients could have been classified as having COPD without the correct spirometry assessment, even though this investigation is formally required for a clinical diagnosis. Thus, the misdiagnosis of COPD could have been occurred for some participants resulting in under- or over-diagnosis. Indeed, in the first years of follow-up, the number of COPD events was under-diagnosed, and the observed results were attenuated; thus, we introduced the 4-year lag period to handle with this effect. Moreover, it is possible that using other criteria for COPD diagnosis could alter the results. In a recently published cross-sectional Danish study, low intake of fruits and raw vegetables was associated with an increased risk of COPD; the risk estimates were lower using the fixed FEV1/FVC ratio < 0.70 plus respiratory symptoms diagnosis compared to those using the lower limit of normal (LLN) criteria to define a diagnosis COPD in spite of a higher number of defined COPD cases [43].

Our study strengthens and expands previous evidence that, in addition to smoking cessation, intake of dietary fiber as a modifiable lifestyle factor may reduce the risk of COPD for both ex- and current long-term smokers. Therefore, our results support the recommendation to increase consumption of products high in dietary fiber especially among smokers. Furthermore, these findings indicate the need to conduct intervention studies on the clinical utility of recommending intake of at least 25 g of fiber/day to reduce the risk of COPD among long-term smokers.

## Electronic supplementary material

Below is the link to the electronic supplementary material.

Supplementary material 1 (PDF 156 kb)
