# Supplementary material for: Long-term dietary fiber intake and risk of chronic obstructive pulmonary disease: a prospective cohort study of women
Source: Eur J Nutr. 2019 Jul 6;59(5):1869–79. doi: 10.1007/s00394-019-02038-w (PMC7351821; doi:10.1007/s00394-019-02038-w)
Supplement: Supplementary file 1 — Supplementary material 1 (PDF 156 kb) [file 394_2019_2038_MOESM1_ESM.pdf]

SUPPLEMENTARY MATERIAL

Long-term dietary fiber intake and risk of COPD: a prospective cohort study of women

Maria Karolina Szmidt[1*], Joanna Kałuża[1,2], Holly Ruth Harris[3,4], Anders Linden[5,6], Alicja Wolk[2,7]

***Corresponding author:** Maria Karolina Szmidt, Department of Human Nutrition, Warsaw University of Life Sciences – SGGW, 159C Nowoursynowska Str., 02-776 Warsaw, Poland, E-mail: maria_szmidt@sggw.pl, telephone number: +48 601-812-660

Start of follow-up

In the current study follow-up started on January 1, 2002 and ended on December 31, 2014. After examining the distribution of annual COPD incidents from 1998 to 2014 (Supplementary Figure 1), the first 4 years of follow-up (1998-2001) were lagged from the analysis, because of probable under-diagnosis at this time. The number of new diagnosis of COPD included in the analysis was 1,557 (2002-2014), instead of 1,771 (1998-2014).

Additionally, the HRs stratified by time of diagnosis were calculated (Supplementary Table 2). Despite of probable under-diagnosis in a first four years, the HRs of COPD in 1998-2014 were very similar to HRs observed in 2002-2014.

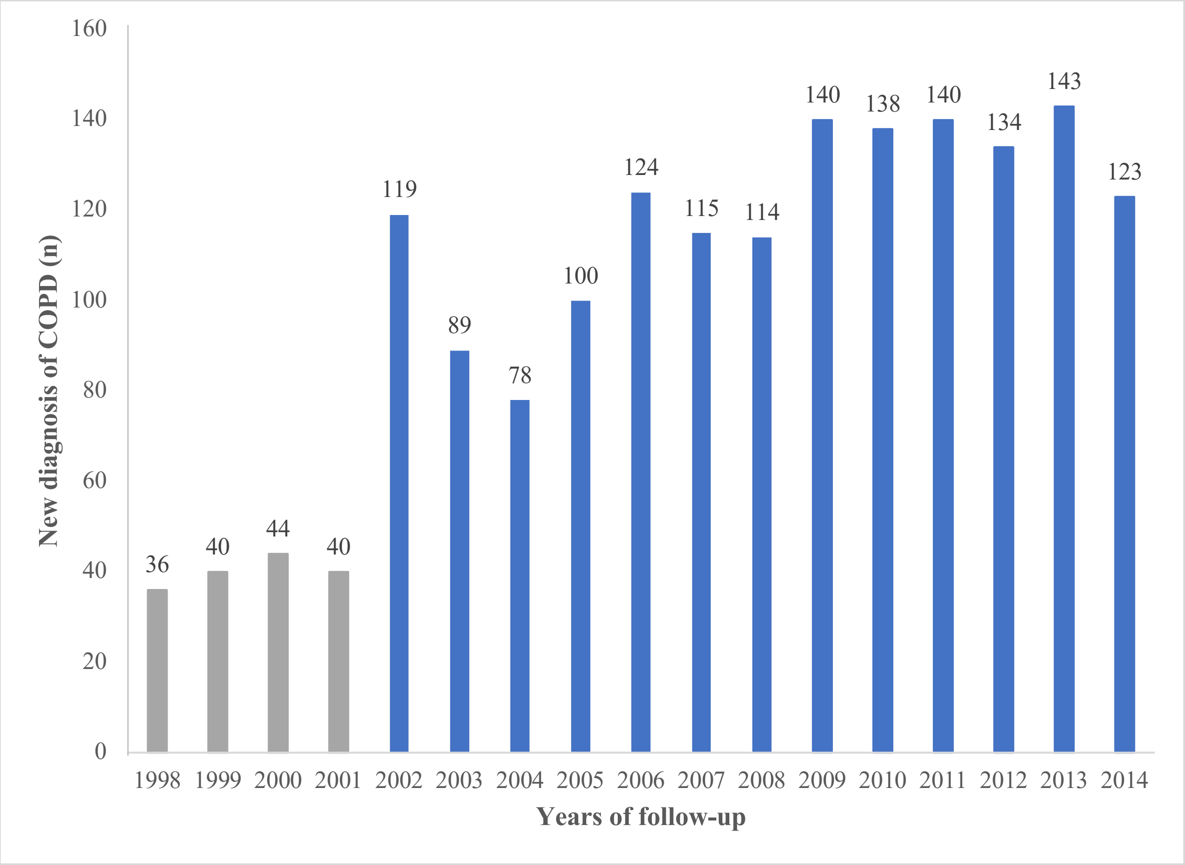

Supplementary Figure S1. Number of cases of new diagnosis of COPD cases identified between 1998 and 2014

Supplementary Table S1. Food items included for each specific dietary fiber calculation in 1987 and 1997

Specific fiber	1987 FFQ	1997 FFQ
**Cereal fiber**	- wholemeal bread<br>- white bread<br>- crisp bread<br>- oat meal/gruel/hot cereal<br>- cold cereal/muesli<br>- rice<br>- spaghetti<br>- pancakes/waffles<br>- rolls/crackers/cookies	- granary/wholemeal bread<br>- white bread<br>- crisp bread<br>- oatmeal porridge<br>- bran of wheat or oats<br>- other porridge/gruel<br>- cereals/muesli<br>- rice<br>- spaghetti/macaroni<br>- pizza<br>- pancakes/crepes<br>- gateau/pastries<br>- buns and cakes<br>- biscuits/wafers/rusks
**Fruit fiber**	- apples/pears<br>- orange/citrus fruits<br>- banana<br>- orange/grapefruit juice<br>- jam/marmalade<br>- sweet soup	- apple/pear<br>- orange/citrus fruits<br>- banana<br>- berries (fresh or frozen)<br>- other fruits<br>- orange/grapefruit juice<br>- jam/marmalade/sauce<br>- stewed fruit/soup
**Vegetable fiber**	- root vegetables<br>- cruciferous vegetables<br>- tomatoes<br>- lettuce/cucumber<br>- spinach/kale	- carrots<br>- beetroots<br>- cabbage<br>- cauliflower<br>- broccoli/Brussels sprouts<br>- tomatoes<br>- lettuce<br>- spinach<br>- peppers<br>- onion/leak<br>- garlic<br>- green peas<br>- mixed frozen vegetables
**Other fiber sources**	- brown beans/pea soup<br>- boiled potatoes	- beans/lentils/pea soup<br>- soy bean products

	- fried potatoes<br>- French fries<br>- chips/popcorn/nuts	- boiled potatoes<br>- fried potatoes<br>- French fries<br>- chips/popcorn<br>- nuts/almonds

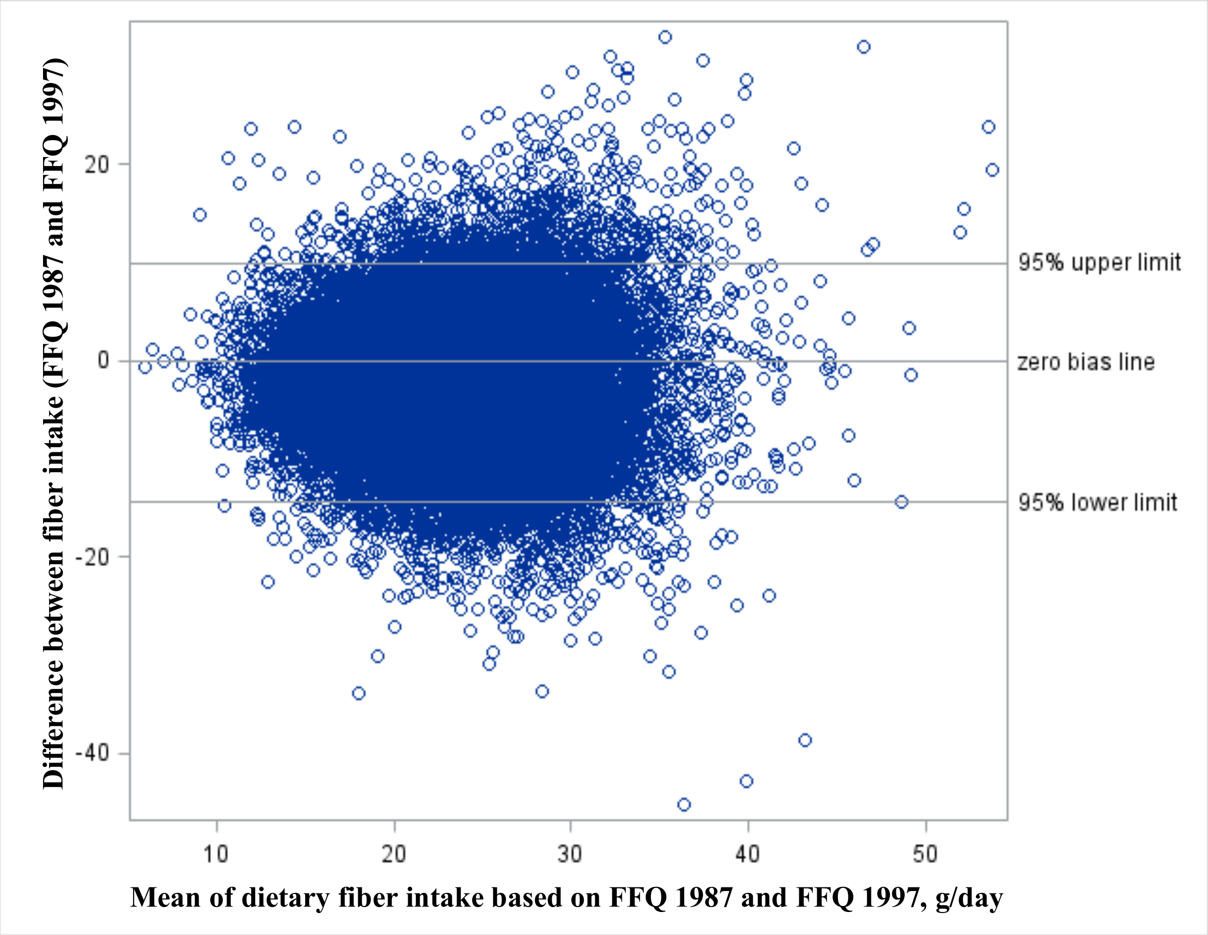

Supplementary Figure S2. Bland-Altman plot[1,2] comparing data on total dietary fiber intake collected using FFQ 1987 and FFQ 1997

References

[1] Bland, J. M. & Altman, D. G. Statistical methods for assessing agreement between two methods of clinical measurement. *Lancet* **1,** 307–310 (1986).

[2] Bland, J. M. & Altman, D. G. Measuring agreement in method comparison studies. *Stat Methods Med Res* **8,** 135–160 (1999).

Supplementary Table S2. Hazard Ratio (95% CI) of COPD stratified by calendar year of follow-up by quintiles of baseline energy-adjusted total dietary fiber, the Swedish Mammography Cohort

Time of COPD diagnose, years	Quintiles of baseline dietary fiber intake, g/day (median)					Baseline dietary fiber intake up to 25 g/day[a]	
	<17.6 (15.6)	17.6-20.5 (19·2)	20.6-23.1 (21.8)	23.2-26.4 (24.6)	≥26.5 (29.1)	Per 1-gram	*P*-trend
1998-2001 (4 years) n=160							
No. of cases/No. women	44/7311	25/7297	32/7294	27/7288	32/7293		
Multivariable-adjusted HR[a] (95% CI)	1.00	0.68 (0.42-1.17)	0.96 (0.61-1.53)	0.81 (0.54-1.44)	1.02 (0.64-1.63)	0.97 (0.93-1.01)	0.17
2002-2008 (7 years) n=739							
No. of cases/No. women	219/7311	146/7288	149/7294	107/7288	118/7293		
Multivariable-adjusted HR[a] (95% CI)	1.00	0.83 (0.67-1.02)	0.91 (0.73-1.12)	0.69 (0.54-0.87)	0.75 (0.60-0.94)	0.96 (0.94-0.99)	<0.001
**2009-2014 (6 years) n=818**							
No. of cases/No. women	227/7311	168/7288	141/7294	141/7288	141/7293		
Multivariable-adjusted HR[a] (95% CI)	1·.00	0.86 (0.70-1.05)	0.77 (0.62-0.96)	0.79 (0.64-0.98)	0.81 (0.65-1.00)	0.97 (0.95-0.99)	0.003

[a]Adjusted for age (continuous), education (less than high school, high school, or university), BMI (<18.5; 18.5-24.9; 25.0-29.9, or ≥30.0 kg/m^2), walking or cycling (<20, 20-60, >60 min/day), smoking status and pack-years of smoking (never; past <20, 20-39, or ≥40 pack-years; or current <20, 20-39, or ≥40 pack-years), intake of energy (kcal/day, quintiles) and alcohol consumption (g/day, quintiles).
